# Protein features fusion using attributed network embedding for predicting protein-protein interaction

**DOI:** 10.1186/s12864-024-10361-8

**Published:** 2024-05-13

**Authors:** Mei-Yuan Cao, Suhaila Zainudin, Kauthar Mohd Daud

**Affiliations:** Center for Artificial Intelligence Technology (CAIT), Faculty of Information Science and Technology, Universiti Kebangsaan Malaysia, Bangi, 43600 Selangor Malaysia

**Keywords:** Protein-protein interaction prediction, Protein sequences, Feature fusion learning, Gaussian kernel, Levenshtein distance

## Abstract

**Background:**

Protein-protein interactions (PPIs) hold significant importance in biology, with precise PPI prediction as a pivotal factor in comprehending cellular processes and facilitating drug design. However, experimental determination of PPIs is laborious, time-consuming, and often constrained by technical limitations.

**Methods:**

We introduce a new node representation method based on initial information fusion, called FFANE, which amalgamates PPI networks and protein sequence data to enhance the precision of PPIs’ prediction. A Gaussian kernel similarity matrix is initially established by leveraging protein structural resemblances. Concurrently, protein sequence similarities are gauged using the Levenshtein distance, enabling the capture of diverse protein attributes. Subsequently, to construct an initial information matrix, these two feature matrices are merged by employing weighted fusion to achieve an organic amalgamation of structural and sequence details. To gain a more profound understanding of the amalgamated features, a Stacked Autoencoder (SAE) is employed for encoding learning, thereby yielding more representative feature representations. Ultimately, classification models are trained to predict PPIs by using the well-learned fusion feature.

**Results:**

When employing 5-fold cross-validation experiments on SVM, our proposed method achieved average accuracies of 94.28%, 97.69%, and 84.05% in terms of Saccharomyces cerevisiae, Homo sapiens, and Helicobacter pylori datasets, respectively.

**Conclusion:**

Experimental findings across various authentic datasets validate the efficacy and superiority of this fusion feature representation approach, underscoring its potential value in bioinformatics.

## Background

The principles of protein-protein interactions (PPIs) involve various aspects such as physical and chemical interactions, molecular recognition mechanisms, and dynamic regulation in living organisms [1]. PPIs are crucial for various biological processes and can be categorized as permanent or brief interactions. Permanent interactions form stable complexes, while brief interactions are dynamic and reversible [2, 3]. Proteins have specific recognition motifs that allow them to interact selectively with their target proteins [4]. Understanding PPIs is vital for unraveling biological processes, identifying therapeutic targets, and developing drugs to modulate specific interactions [5, 6].

Performing biological experiments for detecting PPIs is the most common way to observe how they function. With the development of biological techniques, more PPI data have been collected from high-throughput experiments such as protein chips, yeast two-hybrid (Y2H) systems, mass spectrometry protein complex identification (MS-PCI), and others [4, 7–9]. Nevertheless, carrying out the biological experiment methods is costly, labor-intensive, and has a long cycle [10].

Proteins within cells form complex signaling networks through interactions, which govern crucial aspects such as the cell’s lifecycle, metabolic pathways, and signal transduction [11]. Thanks to advancements in high-throughput experimental methods, such as mass spectrometry analysis and protein interactomics, it has become easier to access a large amount of PPI data [12]. These cutting-edge technologies have facilitated the accumulation of extensive PPI data, which serves as the foundation for predictive research. By integrating and analyzing this wealth of data, we can construct comprehensive protein-protein interaction networks that enable us to gain deeper insights into the essence of protein function and cellular processes [13]. Moreover, these PPI datasets not only provide valuable resources for experimental validation but also serve as crucial training and evaluation benchmarks for the development of prediction models and algorithms [14].

Recently, numerous computational methods have been developed to predict protein-protein interactions (PPIs), which play a crucial role in understanding biological processes and diseases [15–21]. These methods aim to generate prediction results with high confidence, facilitating further research on PPIs. For instance, Wang et al. (2019) proposed a deep learning-based method achieving a high accuracy of 97.31% in a human-related dataset [16]. Computational approaches, such as deep learning and graph-based representations, learn patterns from existing data to predict interactions accurately, thus improving the efficiency and precision of biological experiments. Jha et al. integrated protein sequence-derived features with graph-based representations using Graph-BERT encoding, while Huang et al. introduced SGPPI, a structure-based deep learning framework leveraging AlphaFold2’s monomer structures and graph convolutional networks [21]. TAGPPI is another novel framework utilizing protein sequence data alone, outperforming existing methods and marking the first utilization of predicted protein topology structure graphs for sequence-based PPI prediction [22]. Additionally, PASNVGA utilizes a variational graph autoencoder to integrate sequence and network information, demonstrating superior performance across multiple datasets [23]. DensePPI, proposed by Halsana et al., utilizes a deep convolutional strategy to predict PPIs with high accuracy across diverse organism datasets [24].

Furthermore, protein language models, such as ESM-2 and AlphaFold2, represent a significant advancement in computational biology [25–27]. These models leverage deep learning techniques to predict protein structures directly from primary sequences. ESM-2, a transformer-based protein language model trained on a vast amount of protein sequence data, infers protein structures with remarkable accuracy. Similarly, AlphaFold2 excels in predicting structures from multiple sequence alignments, showcasing the potential of language models to generate accurate structure predictions.

In this research, we introduce an initial information fusion-based node representation method for protein feature presentation by using sequence and interaction network profiles. Specifically, we utilize a Gaussian-kernel-based similarity metric and the Levenshtein distance metric effectively to capture the protein interaction profile and protein sequence information, respectively. To obtain an initial information matrix, a weighted features fusion technique is applied to balance the weight between the two types of information with a weighting parameter. Subsequently, we train a Stacked Autoencoder (SAE) model on the initial information fusion matrix to represent the features of proteins. Finally, an SVM classifier is employed for downstream prediction tasks. To thoroughly assess the performance of our method, we conducted experiments on three commonly used datasets by utilizing a 5-fold cross-validation strategy as used in [28–32]. Notably, our proposed method achieved average accuracies of 94.28%, 97.69%, and 84.05% in terms of *Saccharomyces cerevisiae*, *Homo sapien*, and *Helicobacter pylori* datasets, respectively. Our results demonstrated the effectiveness of this approach by conducting performance comparisons with previous models.

## Results

**Fig. 1:**
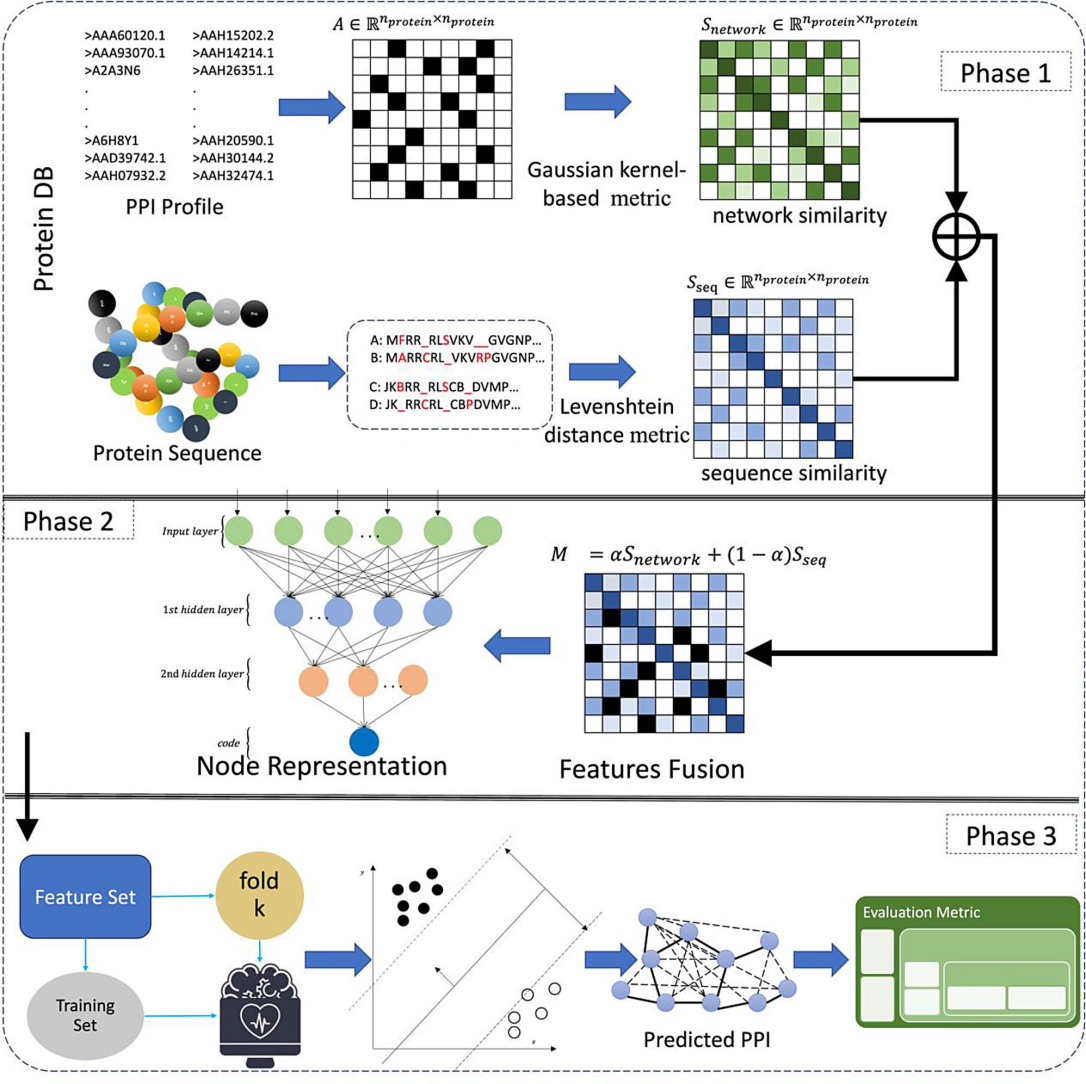
Schematic representation of the proposed methodology

In this study, we propose to employ a feature fusion method for feature learning and a binary classifier for predicting PPIs. Figure 1 shows the overall procedure for the methodology proposed in this research.

This methodology provides a systematic approach for protein-protein interaction prediction, involving data preparation, feature fusion, node embedding, classification model selection and training, and performance evaluation. It offers a framework for accurately predicting protein interactions, thereby contributing to the understanding of biological processes.

In addition, this study utilizes different hyper-parameter alpha for feature fusion learning to obtain new features. The effectiveness of these features is then examined using SVM as a classifier, comparing accuracies to select the optimal parameter settings. These features are considered optimal numerical representations of protein node characteristics, suitable for subsequent classification tasks. By training more complex and robust classifiers, improved classification performance can be achieved.

To evaluate model performance, evaluation metrics are used, which serve as widely adopted and standardized benchmarks for assessing model effectiveness, including accuracy (Acc.), precision (Prec.), sensitivity (Sen.), F1 score, Matthew correlation coefficient (MCC), receiver operating characteristic (ROC) curve, and area under curve of ROC (AUC). Accuracy measures the proportion of correctly classified instances, precision assesses the accuracy of positive predictions, sensitivity indicates the model’s ability to correctly identify positive instances, and the F1 score provides a balance between precision and recall. The MCC considers all four confusion matrix parameters and offers a balanced measure even when classes are of different sizes. Additionally, the ROC curve illustrates the performance of a binary classifier system at various threshold settings, with AUC representing the overall classifier performance. Specifically, an AUC of 1 represents a perfect classifier that correctly ranks all positive instances higher than negative ones, while an AUC of 0.5 suggests a classifier performing no better than random chance.

### Parameter selection of FFANE

In our proposed method for feature fusion learning, there is one parameter for balancing the weight between PPI network information and protein sequence information. From the definition of formula (3), parameter $\alpha$ ranges from 0 to 1. When the parameter $\alpha$ is set to 0.5, it signifies an equal weighting of the two types of information in the features fusion matrix. When $\alpha$ is set to 0, it implies that the features fusion matrix contains only sequence information. Conversely, when $\alpha$ is set to 1, it indicates that the features fusion matrix exclusively comprises network information.

Here, a grid search approach is employed to obtain the best parameter $\alpha$. The parameter $\alpha$ is set to values ranging from 0 to 1, with intervals of 0.125. Upon establishing the parameter $\alpha$ configurations, we proceeded to train the SAE model to learn features corresponding to protein nodes’ features fusion matrix. These extracted features were subsequently subjected to partitioning via a five-fold cross-validation methodology. The SVM classifier was employed as the downstream classification task.

**Fig. 2:**
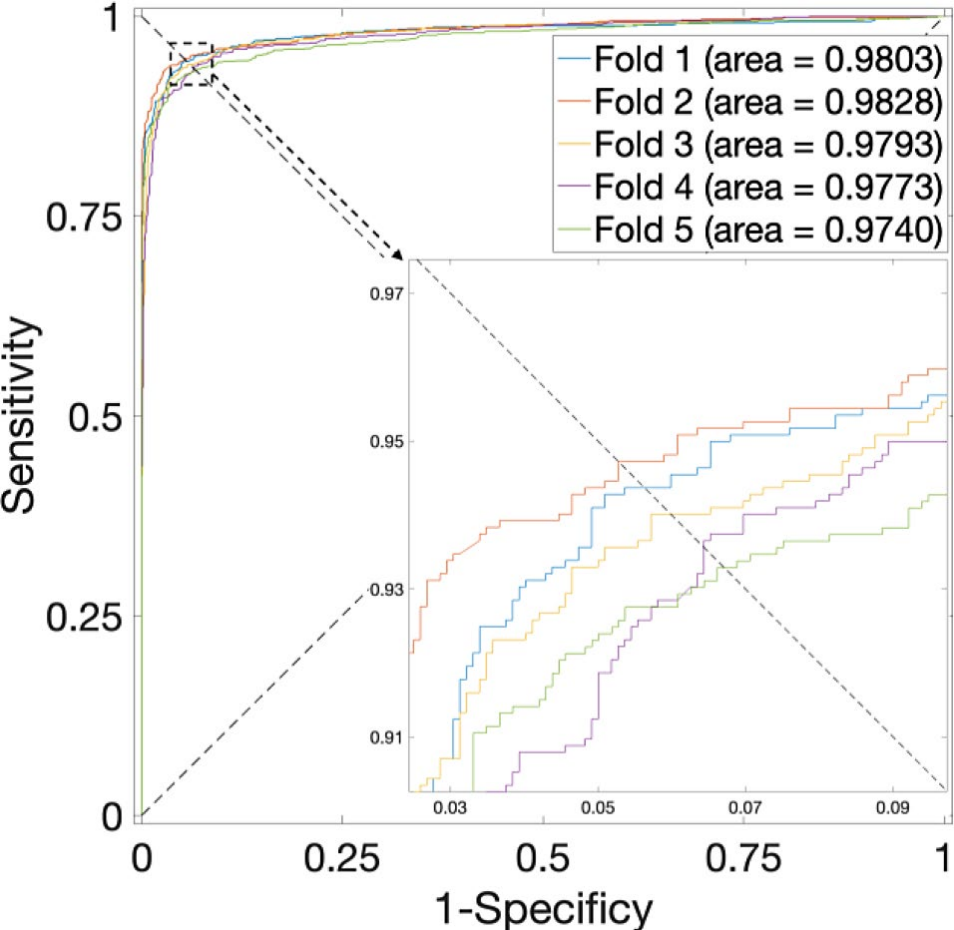
ROC curves for the *S. cerevisiae* Dataset by using SVM and SAE model on features fusion matrix with alpha at 0.375 via 5-Fold CV

Specifically, an in-depth analysis of the outcomes presented in Table 1, particularly concerning the *S. cerevisiae* Dataset, reveals a noteworthy pattern. The highest average accuracy, recorded at 94.28% with a standard deviation of 0.65%, materializes when the parameter $\alpha$ assumes a value of 0.375—noted that the weightage allocated to sequence information stands at 0.625. Corresponding, the ROC curves are plotted in Fig. 2, in which the AUCs are closer to 1 indicating the performance is more powerful. Significantly, when $\alpha$ is set to 0, denoting the exclusion of PPI interaction information in favor of sole reliance on sequence data, the average accuracy experiences a reduction, plummeting to 88.63%. Conversely, when $\alpha$ equals 1, the average accuracy reaches 93.37%.

**Fig. 3:**
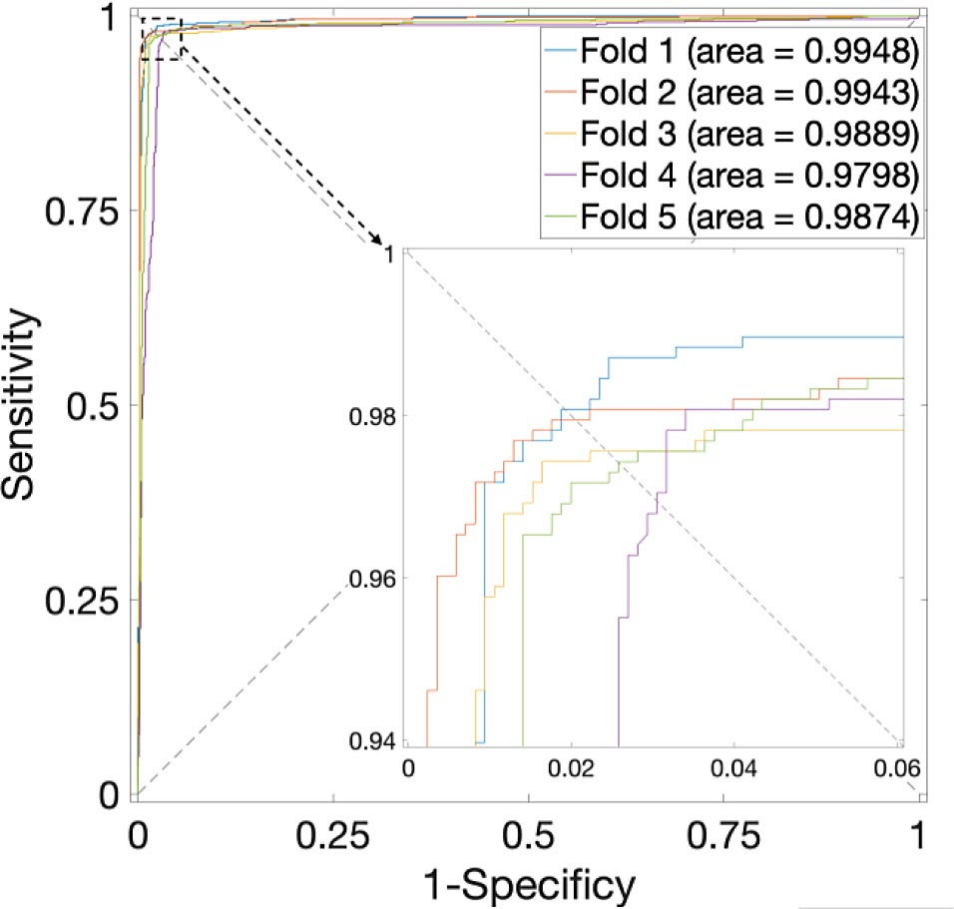
ROC curves for the *H. sapiens* Dataset by using SVM and SAE model on features fusion matrix with alpha at 0.625 via 5-Fold CV

When employing the proposed method on the *H. sapiens* Dataset, as listed in Table 2, the overall average accuracy consistently exceeds 97%, with the highest average accuracy when $\alpha$ is at 0.625. Correspondingly, the ROC curves are plotted in Fig. 3, in which the value of AUC is close to 1. The performance is near perfect.

**Fig. 4:**
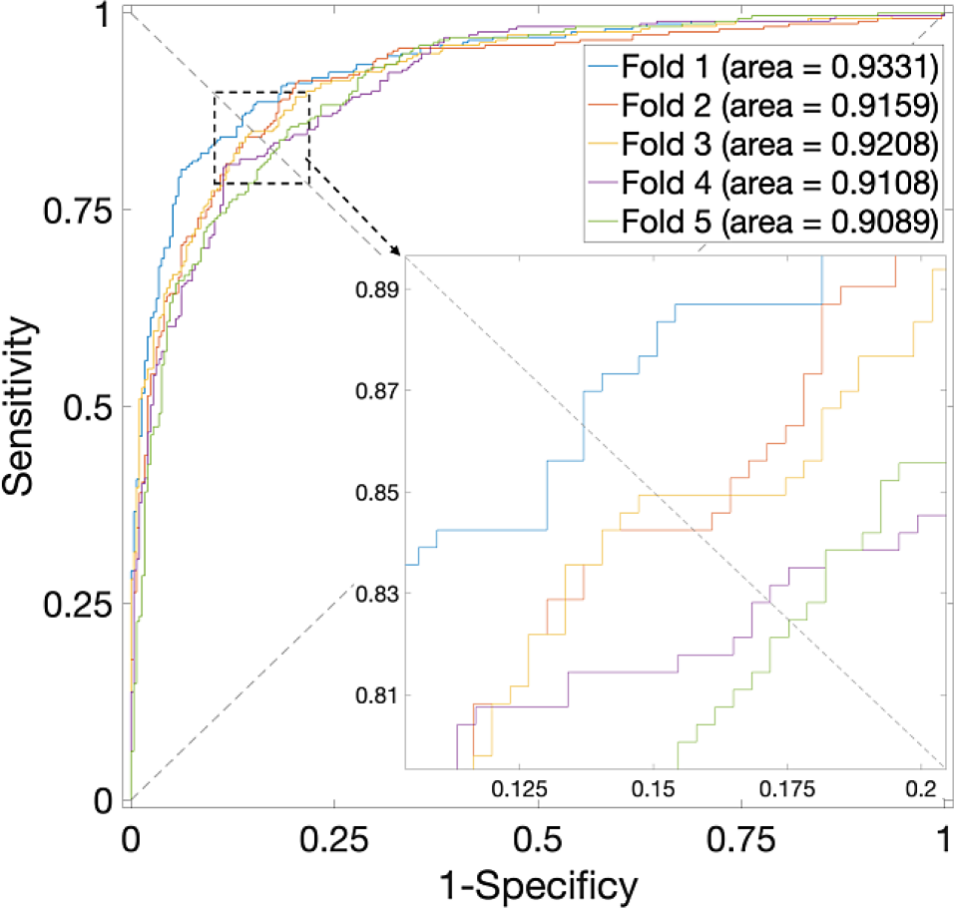
ROC curves for the *H. pylori* Dataset by using SVM and SAE model on features fusion matrix with alpha at 0.75 via 5-Fold CV

Investigations concerning the *H. pylori* Dataset, as detailed in Table 3, unveil a peak average accuracy of 84.05% when $\alpha$ is set to 0.75. $\alpha$ values of 0 or 1 yield average accuracies that fall below 82.58%. Correspondingly, the ROC curves are plotted in Fig. 4, in which the value of the average AUC is 0.9179. The performance is effective.

From the above results, it is evident that FFANE exhibits stronger predictive performance when the alpha parameter is neither 0 nor 1, indicating that the fusion of information outperforms single-source features.

**Table 1:** Prediction results for the *S. cerevisiae* Dataset by using SVM and SAE model on features fusion matrix with different parameter $\alpha$ via 5-fold CV

Test Set	0	0.125	0.25	0.375	0.5	0.625	0.75	0.875	1
Fold 1	88.61	94.91	94.50	94.50	94.95	94.77	93.43	94.46	93.97
Fold 2	88.74	94.95	94.86	95.22	95.13	94.91	95.04	93.70	94.91
Fold 3	88.87	93.88	94.33	94.33	94.01	93.52	93.57	77.12	92.67
Fold 4	87.35	93.61	93.70	93.52	93.48	93.34	93.39	92.98	92.98
Fold 5	89.58	93.83	93.65	93.83	93.74	93.83	94.14	92.89	92.31
Avg.	88.63 ± 0.81	94.23 ± 0.64	94.21 ± 0.52	**94.28 ± 0.65**	94.26 ± 0.74	94.07 ± 0.72	93.91 ± 0.70	90.23 ± 7.36	93.37 ± 1.06

**Table 2:** Prediction results for the *H. sapiens* Dataset by using SVM and SAE model on features fusion matrix with different parameter $\alpha$ via 5-fold CV

Test Set	0	0.125	0.25	0.375	0.5	0.625	0.75	0.875	1
Fold 1	76.61	97.92	98.22	97.73	98.16	98.04	98.47	98.35	98.65
Fold 2	83.71	96.75	92.84	97.8	97.43	98.22	97.37	95.77	97.86
Fold 3	81.92	95.77	96.94	97.49	96.81	97.86	95.22	97.06	97.3
Fold 4	76.47	97.49	97.49	97.49	97.37	96.81	97.61	97.61	90.38
Fold 5	79.71	97.73	97	97.36	95.89	97.49	97.55	97.24	97.49
Avg.	79.68 ± 3.2	97.13 ± 0.88	96.5 ± 2.11	97.57 ± 0.18	97.13 ± 0.84	**97.69 ± 0.56**	97.24 ± 1.21	97.21 ± 0.94	96.34 ± 3.37

**Table 3:** Prediction results for the *H. pylori* Dataset by using SVM and SAE model on features fusion matrix with different parameter $\alpha$ via 5-fold CV

Test Set	0	0.125	0.25	0.375	0.5	0.625	0.75	0.875	1
Fold 1	77.4	83.9	84.93	85.27	85.27	84.93	86.64	82.88	86.3
Fold 2	78.42	85.27	84.08	83.56	83.9	82.02	84.25	85.1	84.25
Fold 3	74.83	83.05	83.39	83.22	82.53	83.56	84.59	84.59	79.45
Fold 4	78.01	83.68	82.13	81.96	80.93	81.62	82.82	82.65	80.76
Fold 5	76.63	82.3	82.47	83.68	83.51	83.85	81.96	83.85	82.13
Avg.	77.06 ± 1.42	83.64 ± 1.1	83.4 ± 1.15	83.54 ± 1.18	83.23 ± 1.62	83.2 ± 1.36	**84.05 ± 1.8**	83.81 ± 1.06	82.58 ± 2.74

### Prediction performance among different classifiers

In this section, some classic classifiers are trained, including XGBoost(XGB), Random Forest(RF), Naïve Bayes(NB). For the *S. cerevisiae* Dataset, *H. sapiens* Dataset, and *H. pylori* Dataset, the parameters for alpha in FFANE were set to 0.375, 0.625, and 0.75, respectively.

Tables 4 and 5, and 6 present the experimental results of our feature fusion method combined with various classifiers on three datasets. The experimental outcomes illustrate that the accuracy of the feature fusion method combined with the XGB classifier surpasses that of the other three approaches.

**Fig. 5:**
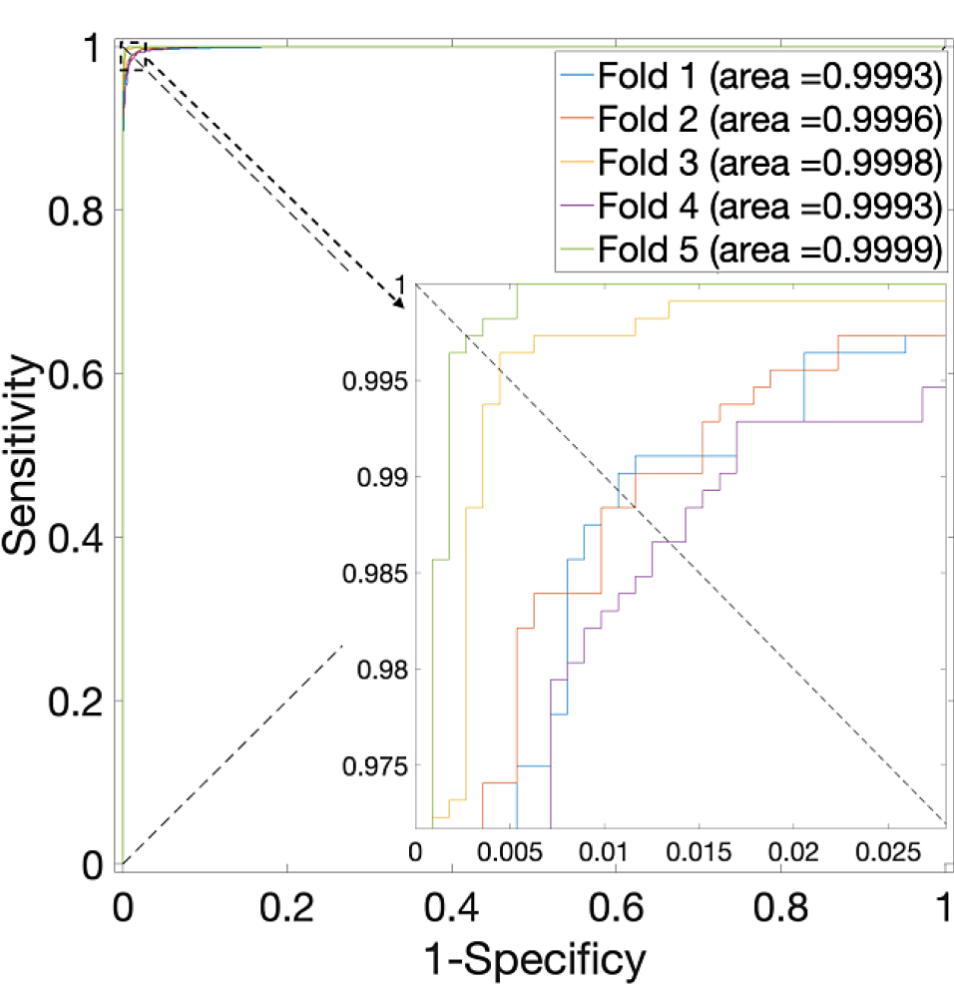
ROC curves for the *S. cerevisiae* Dataset by using XGBoost and SAE model on features fusion matrix with alpha at 0.375 via 5-Fold CV

**Fig. 6:**
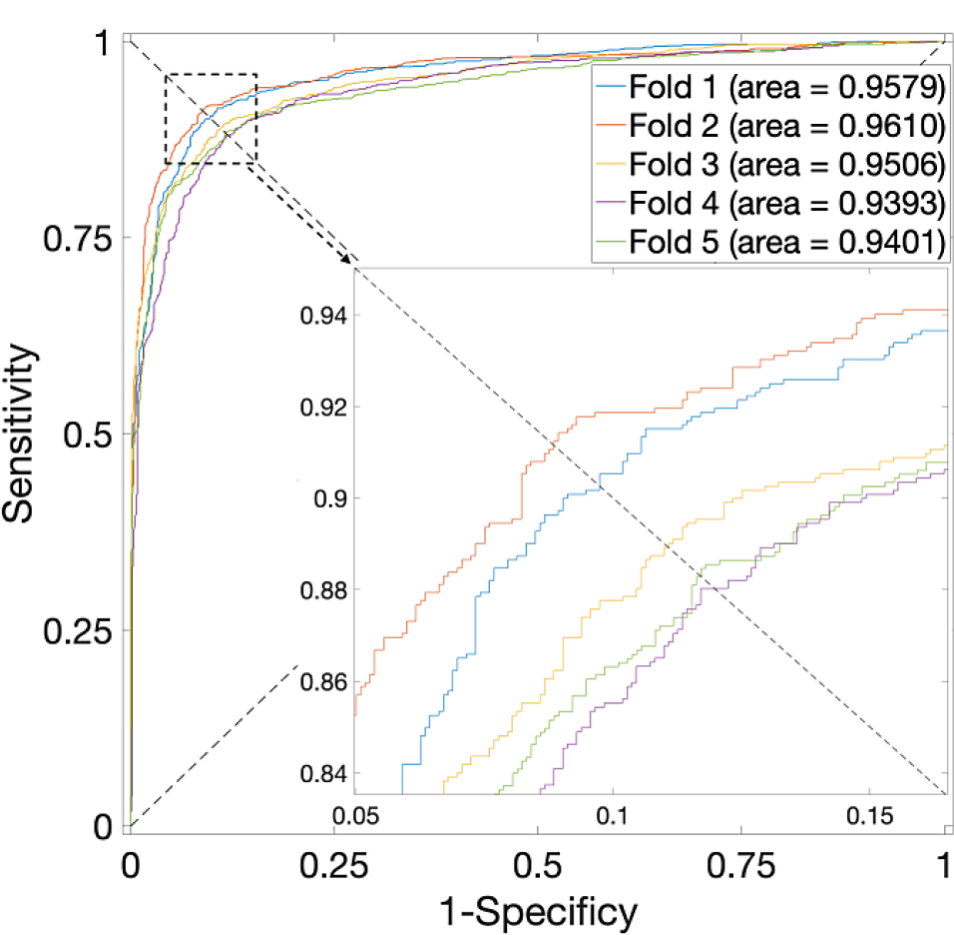
ROC curves for the *S. cerevisiae* Dataset by using NB and SAE model on features fusion matrix with alpha at 0. 375 via 5-Fold CV

**Fig. 7:**
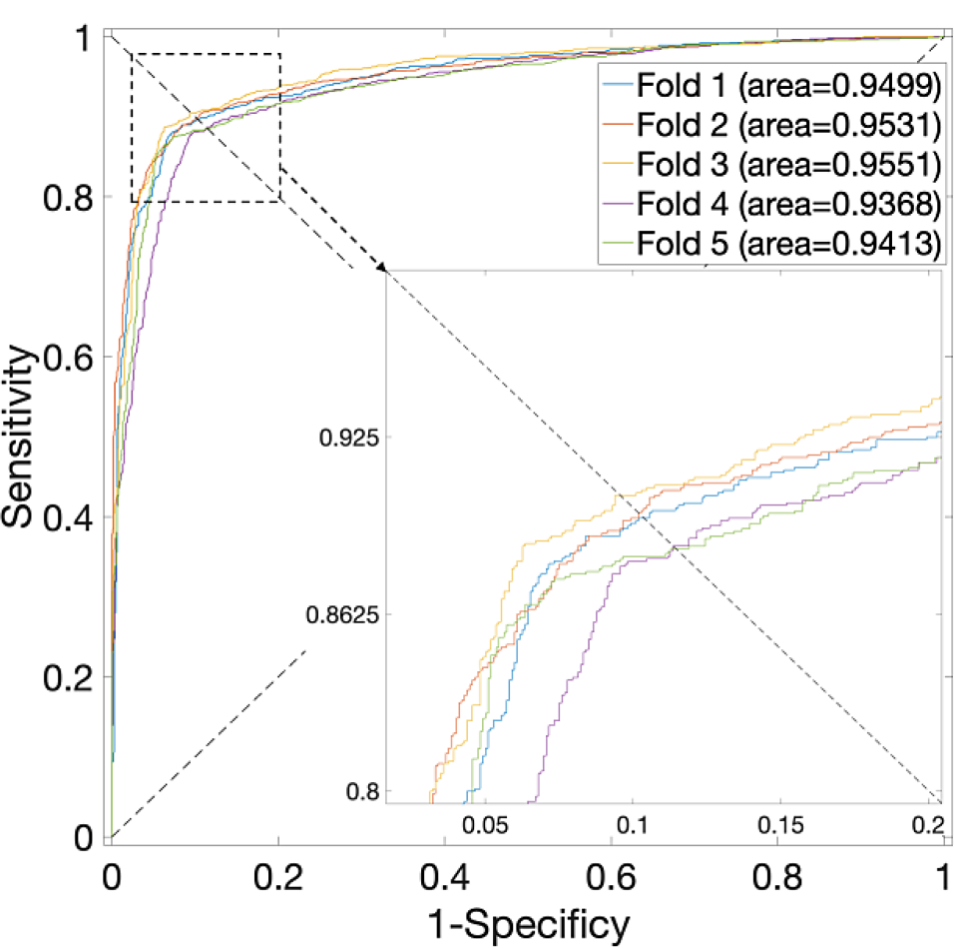
ROC curves for the *S. cerevisiae* Dataset by using RF and SAE model on features fusion matrix with alpha at 0.375 via 5-Fold CV

In Table 4, for the *S. cerevisiae* dataset, the use of the XGB classifier resulted in a 5.07% accuracy improvement over the SVM classifier, a 97.79% improvement over the RF classifier and an 11.92% improvement over the NB classifier. The corresponding ROC of XGB, RF and NB is plotted in Figs. 5 and 6, and Fig. 7, respectively.

**Table 4:** Prediction results of 5-fold CV for the *S. cerevisiae* Dataset by using different classifiers

Classifiers	Test Set	Acc. (%)	Prec. (%)	Sens. (%)	F1 score (%)	MCC	AUC
SVM	Fold 1	94.55	94.87	94.19	94.53	0.891	0.9803
	Fold 2	95.13	96.5	93.66	95.06	0.903	0.9828
	Fold 3	94.24	95.08	93.3	94.18	0.8849	0.9793
	Fold 4	93.39	93.7	93.03	93.36	0.8678	0.9773
	Fold 5	93.65	95.44	91.68	93.52	0.8737	0.974
	Avg.	94.19 ± 0.7	95.12 ± 1.01	93.17 ± 0.94	94.13 ± 0.71	0.8841 ± 0.014	0.9787 ± 0.0033
XGBoost	Fold 1	98.93	99.02	98.84	98.93	0.9786	0.9993
	Fold 2	98.61	99.45	97.77	98.60	0.9724	0.9996
	Fold 3	99.11	99.73	98.48	99.10	0.9822	0.9998
	Fold 4	98.48	99.27	97.68	98.47	0.9697	0.9993
	Fold 5	99.73	99.82	99.64	99.73	0.9946	0.9999
	Avg.	98.97 ± 0.44	99.46 ± 0.29	98.48 ± 0.73	98.97 ± 0.44	0.9795 ± 0.0087	0.9996 ± 0.0003
RF	Fold 1	90.35	92.80	87.49	90.06	0.8083	0.9499
	Fold 2	90.08	93.42	86.24	89.68	0.8040	0.9531
	Fold 3	91.15	93.40	88.56	90.92	0.8242	0.9551
	Fold 4	89.14	89.96	88.11	89.03	0.7830	0.9368
	Fold 5	89.98	92.49	87.03	89.68	0.8010	0.9413
	Avg.	90.14 ± 0.64	92.41 ± 1.28	87.49 ± 0.81	89.87 ± 0.62	0.8041 ± 0.0132	0.9472 ± 0.0070
NB	Fold 1	89.05	93.27	84.18	0.8849	0.7848	0.9579
	Fold 2	89.14	96.3	81.41	0.8823	0.7924	0.9610
	Fold 3	88.11	94.38	81.05	0.8721	0.7700	0.9506
	Fold 4	87.89	89.92	85.34	0.8757	0.7588	0.9393
	Fold 5	87.97	91.01	84.26	0.8751	0.7615	0.9401
	Avg.	88.43 ± 0.61	92.98 ± 2.56	83.25 ± 1.9	0.88 ± 0.01	0.7735 ± 0.0146	0.9498 ± 0.0099

**Fig. 8:**
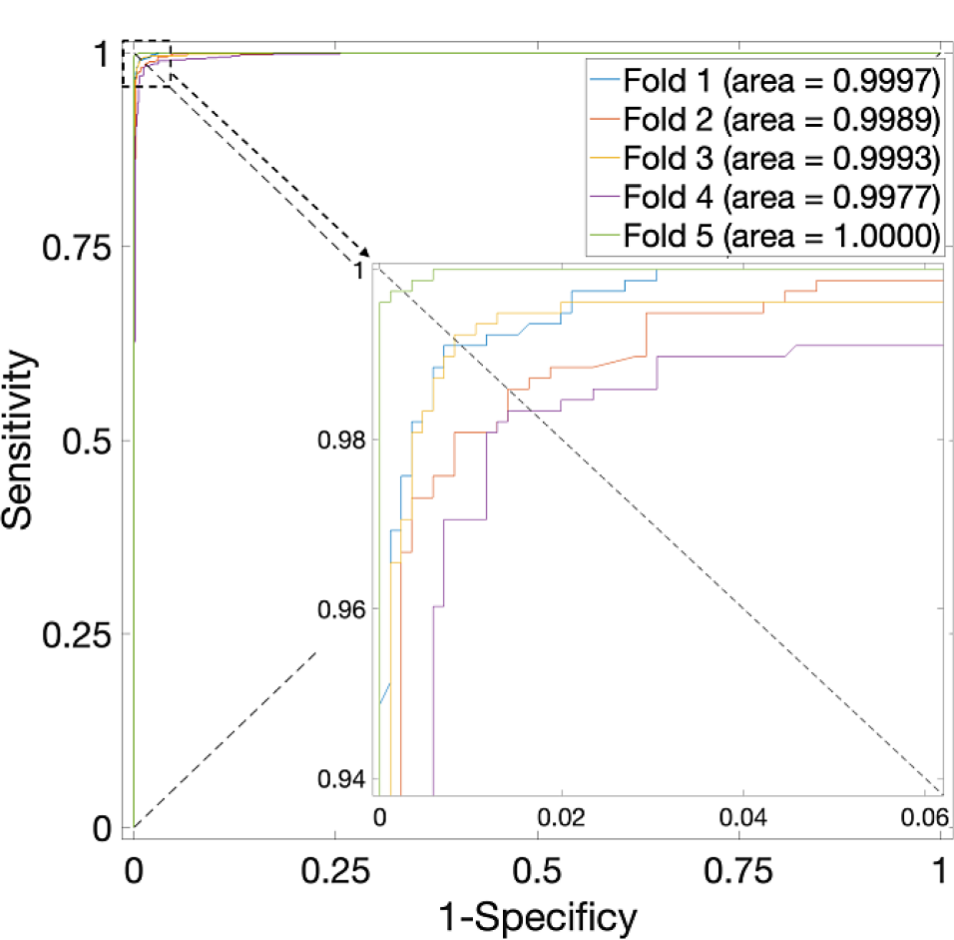
ROC curves for the *H. sapiens* Dataset by using XGBoost and SAE model on features fusion matrix with alpha at 0. 625 via 5-Fold CV

**Fig. 9:**
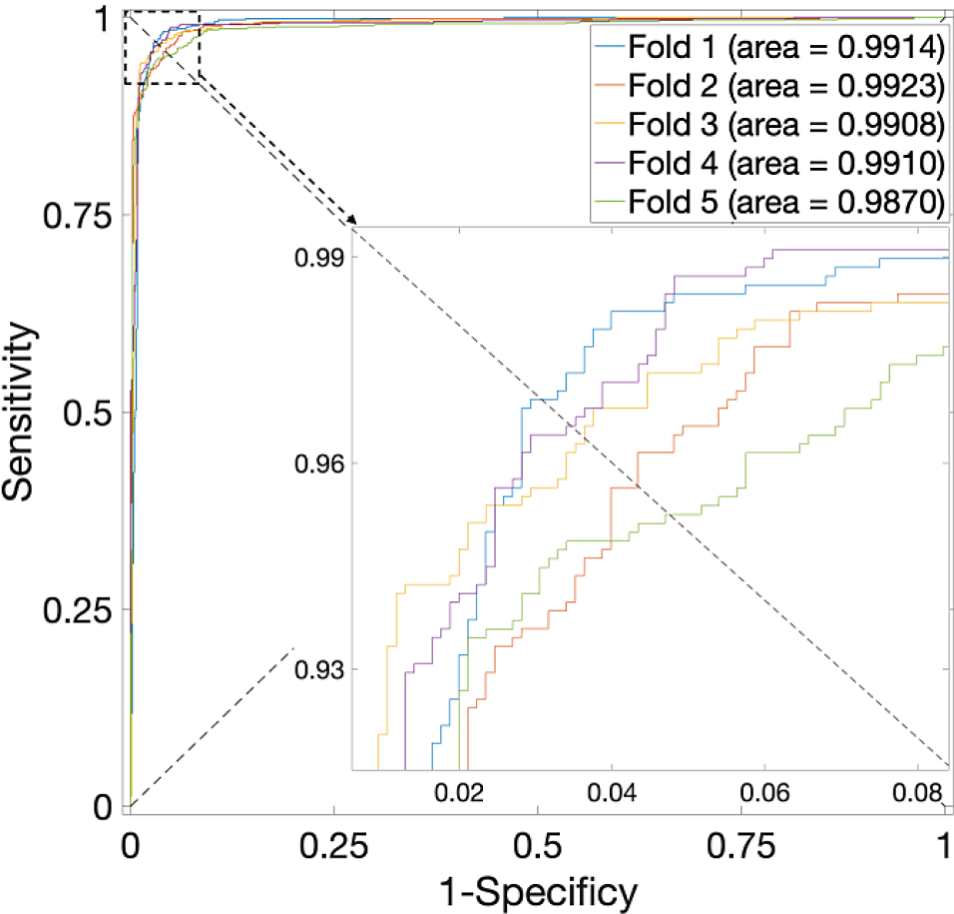
ROC curves for the *H. sapiens* Dataset by using NB and SAE model on features fusion matrix with alpha at 0. 625 via 5-Fold CV

**Fig. 10:**
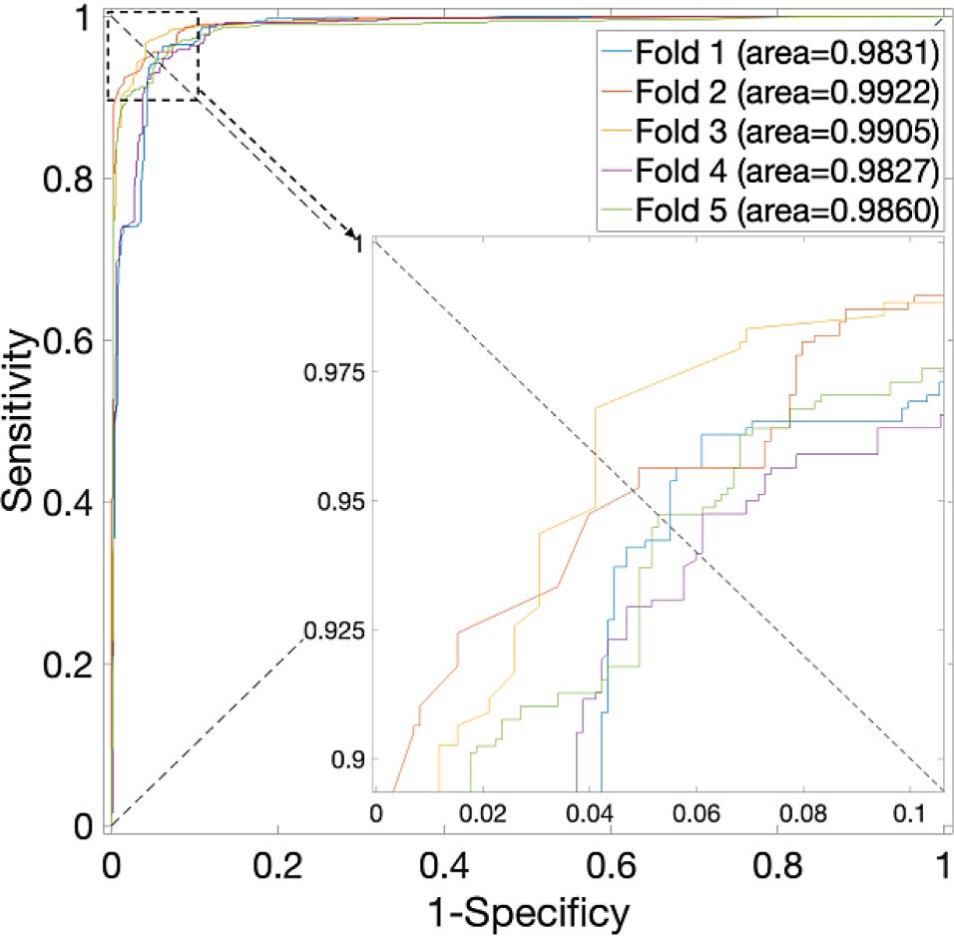
ROC curves for the *H. sapiens* Dataset by using RF and SAE model on features fusion matrix with alpha at 0. 625 via 5-Fold CV

In Table 5, for the *H. sapiens* dataset, FFANE-XGB outperforms FFANE-SVM, FFANE-RF and FFANE-NB by 5.08%, 9.8% and 11.92% in accuracy. The corresponding ROC of XGB, RF and NB is plotted in Figs. 8 and 9, and Fig. 10, respectively.

**Fig. 11:**
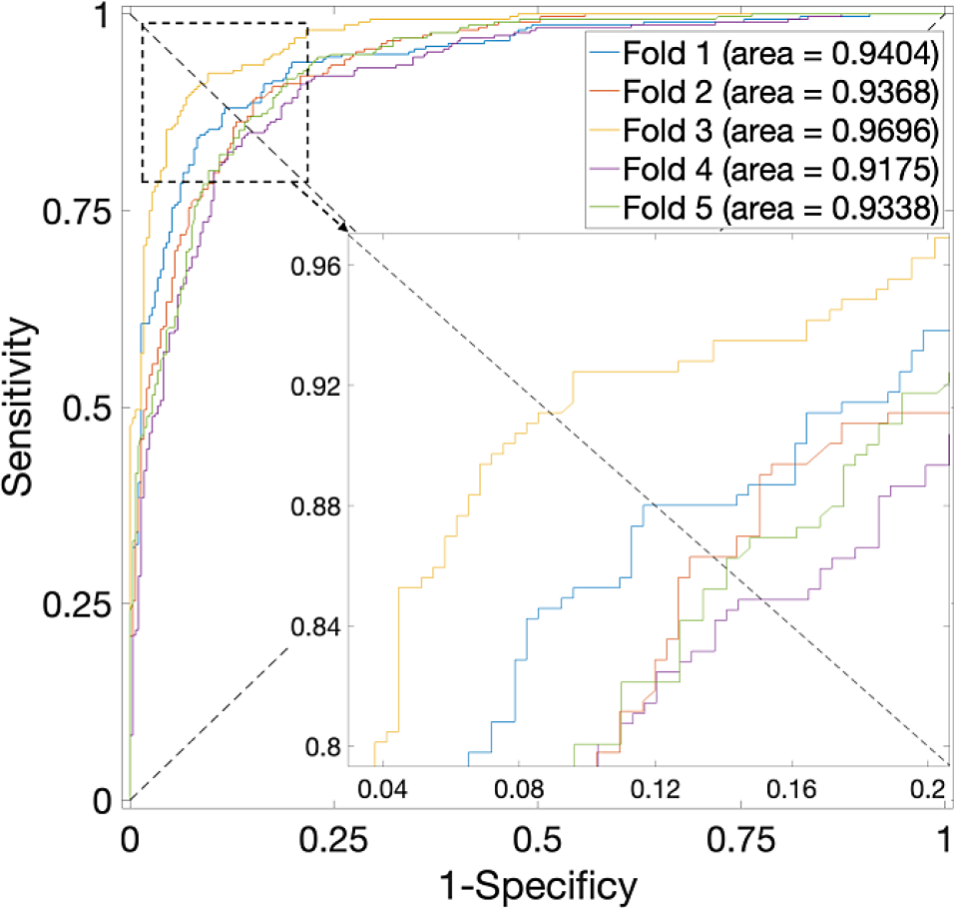
ROC curves for the *H. pylori* Dataset by using XGBoost and SAE model on features fusion matrix with alpha at 0.75 via 5-Fold CV

**Fig. 12:**
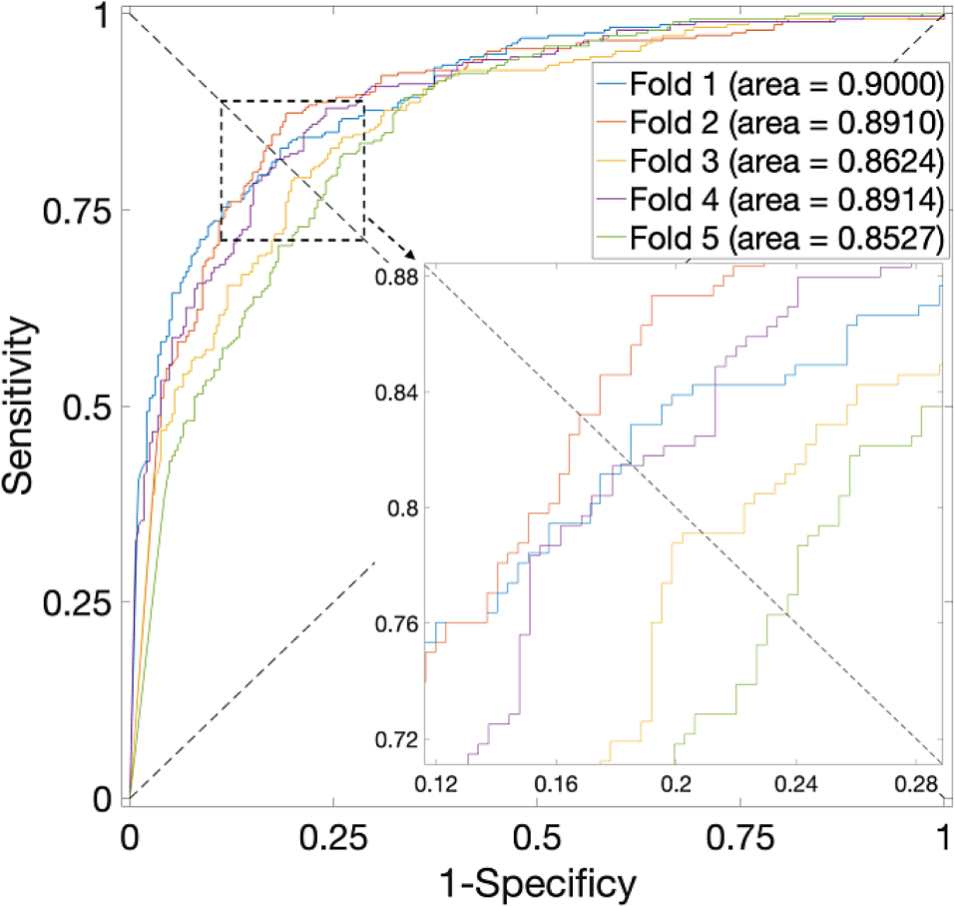
ROC curves for the *H. pylori* Dataset by using NB and SAE model on features fusion matrix with alpha at 0. 75 via 5-Fold CV

**Fig. 13:**
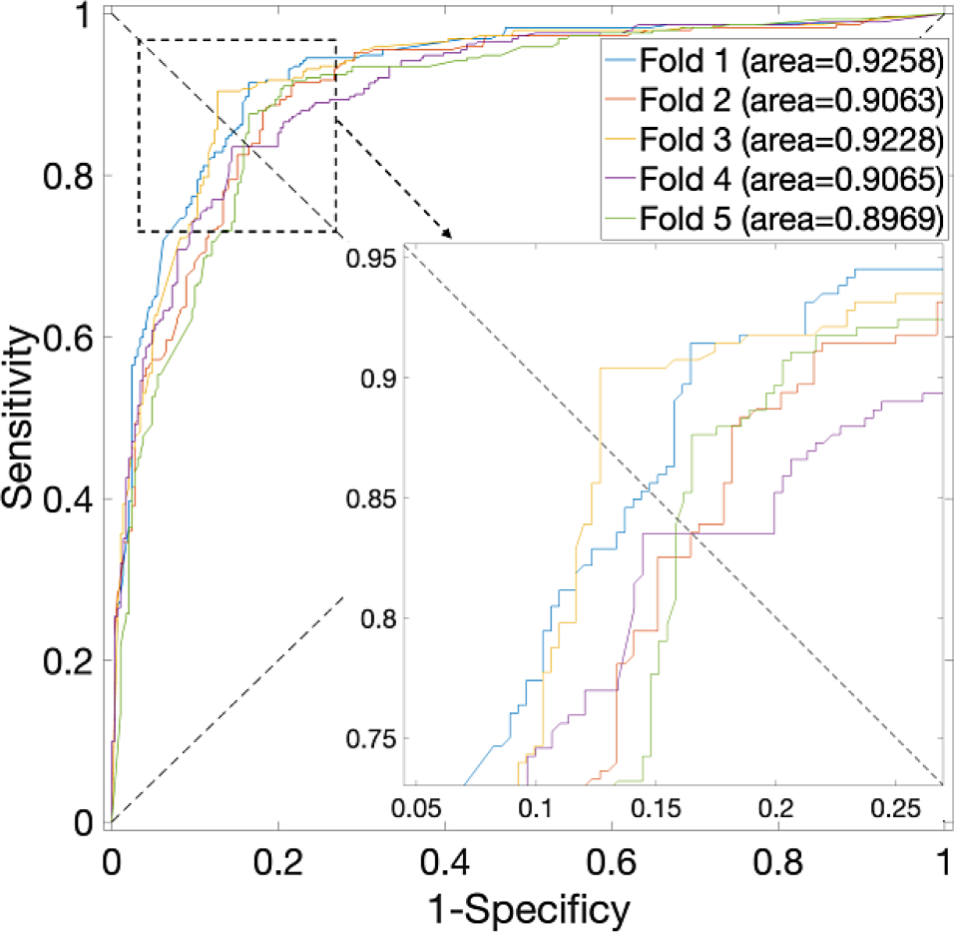
ROC curves for the *H. pylori* Dataset by using RF and SAE model on features fusion matrix with alpha at 0. 75 via 5-Fold CV

In Table 6, a similar trend is observed when applying these methods to the *H. pylori* dataset, where the XGB classifier demonstrates a significant increase in accuracy compared to the other three classifiers. The corresponding ROC of XGB, RF and NB are plotted in Figs. 11 and 12, and Fig. 13, respectively.

These results’ enhancement may be attributed to the fact that the XGBoost classifier is more advanced than the SVM, RF, and NB classifiers. This highlights the prospect of achieving superior results by integrating our feature fusion technique with the latest advancements in classification methods.

**Table 5:** Prediction results of 5-fold CV for the *H. sapiens* Dataset by using different classifiers

Classifiers	Test Set	Acc. (%)	Prec. (%)	Sens. (%)	F1 score (%)	MCC	AUC
SVM	Fold 1	98.16	98.7	97.44	0.9806	0.9632	0.9948
	Fold 2	98.16	99.08	97.05	0.9806	0.9633	0.9943
	Fold 3	97.79	98.69	96.67	0.9767	0.9559	0.9889
	Fold 4	96.81	96.79	96.54	0.9666	0.9361	0.9798
	Fold 5	97.55	97.8	97.05	0.9742	0.9509	0.9874
	Avg.	97.69 ± 0.56	98.21 ± 0.92	96.95 ± 0.36	0.98 ± 0.01	0.9539 ± 0.0112	0.989 ± 0.0061
XGBoost	Fold 1	99.08	98.98	99.1	0.9904	0.9816	0.9997
	Fold 2	98.53	98.84	98.08	0.9846	0.9706	0.9989
	Fold 3	99.14	99.23	98.97	0.9910	0.9828	0.9993
	Fold 4	98.47	98.58	98.21	0.9839	0.9693	0.9977
	Fold 5	99.82	99.87	99.74	0.9981	0.9963	1.0000
	Avg.	99.01 ± 0.55	99.1 ± 0.49	98.82 ± 0.68	0.99 ± 0.01	0.9801 ± 0.011	0.9991 ± 0.0009
RF	Fold 1	92.65	95.08	89.23	92.06	0.8538	0.9831
	Fold 2	93.51	99.71	86.67	92.73	0.8762	0.9922
	Fold 3	92.28	99.25	84.49	91.27	0.8531	0.9905
	Fold 4	88.79	96.28	79.62	87.16	0.7847	0.9827
	Fold 5	89.82	99.68	78.95	88.11	0.8107	0.9860
	Avg.	91.41 ± 1.79	98.00 ± 1.94	83.79 ± 3.98	90.27 ± 2.22	0.8357 ± 0.0332	0.9869 ± 0.0039
NB	Fold 1	95.41	97.7	92.56	0.9506	0.9089	0.9914
	Fold 2	94.67	97.79	90.9	0.9422	0.8950	0.9923
	Fold 3	95.77	98.77	92.31	0.9543	0.9169	0.9908
	Fold 4	94	98.85	88.46	0.9337	0.8837	0.9910
	Fold 5	94.11	98.44	89.09	0.9353	0.8853	0.9870
	Avg.	94.79 ± 0.78	98.31 ± 0.54	90.66 ± 1.85	0.94 ± 0.01	0.8979 ± 0.0146	0.9905 ± 0.002

**Table 6:** Prediction results of 5-fold CV for the *H. pylori* Dataset by using different classifiers

Feature	Test Set	Acc. (%)	Prec. (%)	Sens. (%)	F1 score (%)	MCC	AUC
SVM	Fold 1	86.64	92.13	80.14	0.8571	0.7392	0.9331
	Fold 2	84.08	87.27	79.79	0.8336	0.6840	0.9159
	Fold 3	84.08	87.27	79.79	0.8336	0.6840	0.9208
	Fold 4	83.85	85.56	81.44	0.8345	0.6778	0.9108
	Fold 5	81.62	86.51	74.91	0.8029	0.6381	0.9089
	Avg.	84.05 ± 1.78	87.75 ± 2.55	79.21 ± 2.5	0.83 ± 0.02	0.6846 ± 0.036	0.9179 ± 0.0097
XGBoost	Fold 1	86.99	85.76	88.7	0.8721	0.7402	0.9404
	Fold 2	86.82	85.25	89.04	0.8710	0.7370	0.9368
	Fold 3	91.1	91.1	91.1	0.9110	0.8219	0.9696
	Fold 4	84.54	81.9	88.66	0.8515	0.6931	0.9175
	Fold 5	85.91	83.39	89.69	0.8642	0.7203	0.9338
	Avg.	87.07 ± 2.45	85.48 ± 3.5	89.44 ± 1.02	0.87 ± 0.02	0.7425 ± 0.0482	0.9396 ± 0.0189
RF	Fold 1	87.50	84.76	91.44	87.97	0.7523	0.9258
	Fold 2	84.93	82.90	88.01	85.38	0.7000	0.9063
	Fold 3	88.70	87.67	90.07	88.85	0.7743	0.9228
	Fold 4	84.54	85.26	83.51	84.38	0.6909	0.9065
	Fold 5	85.57	84.16	87.63	85.86	0.7119	0.8969
	Avg.	86.25 ± 1.59	84.95 ± 1.57	88.14 ± 2.70	86.49 ± 1.67	0.7259 ± 0.0320	0.9117 ± 0.0109
NB	Fold 1	81.16	82.27	79.45	0.8084	0.6237	0.9000
	Fold 2	83.73	82.08	86.3	0.8414	0.6755	0.8910
	Fold 3	79.11	76.4	84.25	0.8013	0.5853	0.8624
	Fold 4	81.27	81.16	81.44	0.8130	0.6254	0.8914
	Fold 5	76.98	76.43	78.01	0.7721	0.5396	0.8527
	Avg.	80.45 ± 2.54	79.67 ± 3	81.89 ± 3.4	0.81 ± 0.02	0.6099 ± 0.0507	0.8795 ± 0.0206

### Comparison with state-of-the-art prediction methods

In this section, we compare our proposed method among the existing methods that use different types of fusion approaches based on 5-CV, also see Table 7.

Some use one kind of feature extraction. Li et al. proposed to use Scale-Invariant Feature Transform (SIFT) algorithm method on Position Weight Matrix (PWM) from protein sequences [28]. Position-Specific Scoring Matric (PSSM) involves transforming protein sequences using PSI-BLAST, which is widely employed to extract sequence feature. The original matric cannot be used directly for classifier training as feature vector. To extract features, Li et al. proposed to use the Orthogonal Locality Preserving Projections (OLPP) algorithm that aims to preserve local structure and discriminative information while reducing dimensionality, resulting in fixed-length feature vectors that represent each protein [29].

Some use more than two kinds of feature extraction methods. An et al. proposed PSSM-SVM to fusion two kinds of features via Bigram Probability(BP) and Local Average Group (LAG) on PSSM [33]. AE-SVM model is a predictive model that combines AE and SVM. it leverages sequence information using CT and CTD feature extraction methods [34]. The AE reduces the dimensionality of the features. The functional-link Siamese neural network (FSNN-SVM) uses the fusion of features derived using pseudo amino acid composition and conjoint triad descriptors [30]. The FSNN extracts the high-level abstraction features from the raw features and SVM performs the PPI prediction task using these abstraction features. Wang et al. proposed a novel deep learning algorithm called symmetric nonnegative latent factorization (SNLF) [31]. The method enhances the quality of PPI data using SNLF and encodes proteins using Quasi-Sequence-Order based on their sequence information. Principal component analysis is utilized for compact feature generation, and a graph variational AE learns protein embeddings considering features and network topology. The embeddings are then fed into a feedforward neural network for PPI prediction. StackPPI is proposed to utilize 6 kinds of features and applies XGBoost for feature noise reduction and dimensionality reduction [32]. The optimized features are then analyzed using a stacked ensemble classifier consisting of random forest, extremely randomized trees, and logistic regression algorithms.

In Table 7, it is evident that when our method is applied to the *S. cerevisiae* dataset and the *H. sapiens* dataset, the accuracy of the proposed method (SVM) surpasses that of other existing methods, reaching 94.19% and 97.69%, respectively. This indicates a marked improvement in performance following feature fusion. However, when the proposed method (SVM) is applied to the *H. pylori* dataset, the accuracy drops to 84.05%, slightly lower than the highest accuracy of 88.47% achieved by the FSNN-SVM method. This discrepancy may be attributed to the relatively small size of the *H. pylori* dataset (only 2916 protein interactions), which is prone to overfitting when working with limited protein interaction data. In contrast, the other two datasets are larger, allowing our method to deliver more favorable outcomes. Consequently, our approach is better suited for larger datasets, aligning with the inevitable trend of growing protein interaction datasets as our understanding of protein interactions continues to expand. Additionally, our proposed method (XGB) outperforms proposed method (SVM) across all three datasets, emphasizing the promising advantages of amalgamating our feature fusion approach with state-of-the-art technology. It underscores the potential to attain superior outcomes by integrating our feature fusion technique with the most recent innovations in classification methodologies. We also provide statistical significance test results in Tables 8, 9 and 10 for three datasets by using different methods. The results show that our proposed method combined with XGB and SVM is significantly superior to other methods.

**Table 7:** Performance comparison among the existing methods

Dataset	Feature	Model	ACC%	Sen%	Prec%	MCC
*S. cerevisiae*	Biological Sequence	Bio2Vec	93.3	92.7	93.55	0.8749
	OLPP	OLPP-SVM	78.96 ± 1.55	78.76 ± 2.37	79.08 ± 1.03	0.6680 ± 0.0175
	SIFT	SIFT-SVM	91.27 ± 1.06	92.05 ± 0.55	90.39 ± 1.17	0.8255 ± 0.0211
	PseAAC, CT	FSNN-SVM	87.96	N/A	N/A	N/A
	Network structure, sequence	SNLF + QSO	81.00	N/A	93.00	N/A
	CT, CTD	AE-SVM	93.40 ± 0.20	90.6 ± 0.4	N/A	0.87 ± 0.004
	BP, LAG	PSSM-SVM	90.48 ± 0.76	90.26 ± 0.87	90.58 ± 0.98	0.8284 ± 0.0127
	TAGPPI	(end-to-end)	97.81	98.26	98.10	0.9563
	**network structure, sequence**	**proposed method (SVM)**	94.19 ± 0.7	93.17 ± 0.94	95.12 ± 1.01	0.8841 ± 0.014
	**network structure, sequence**	**proposed method (XGB)**	**98.97 ± 0.44**	**98.48 ± 0.73**	**99.46 ± 0.29**	**0.9795 ± 0.0087**
*H. pylori*	PseAAC, CT	FSNN-SVM	**88.47**	N/A	N/A	N/A
	SIFT	SIFT-SVM	80.49 ± 1.40	82.30 ± 2.72	77.79 ± 2.60	0.6111 ± 0.0273
	Biological Sequence	Bio2Vec-Based	88.01	89.61	99.5	0.7871
	**network structure, sequence**	**proposed method (SVM)**	84.05 ± 1.78	79.21 ± 2.5	87.75 ± 2.55	0.6846 ± 0.036
	**network structure, sequence**	**proposed method (XGB)**	87.07 ± 2.45	89.44 ± 1.02	85.48 ± 3.5	0.7425 ± 0.0482
*H. sapiens*	CT, CTD	AE-SVM	97.30 ± 0.2	95.90 ± 0.3	N/A	0.946 ± 0.004
	Biological Sequence	Bio2Vec-Based	97.31	96.28	98.48	0.9476
	SIFT	SIFT-SVM	96.55 ± 0.71	97.12 ± 0.44	96.15 ± 1.49	0.9311 ± 0.0141
	OLPP	OLPP-SVM	87.23 ± 0.57	87.23 ± 0.58	85.83 ± 1.16	0.7766 ± 0.0087
	**network structure, sequence**	**proposed method (SVM)**	97.69 ± 0.56	96.95 ± 0.36	98.21 ± 0.92	0.9539 ± 0.0112
	**network structure, sequence**	**proposed method (XGB)**	**99.01 ± 0.55**	**98.82 ± 0.68**	**99.1 ± 0.49**	**0.9801 ± 0.011**

**Table 8:** Results of statistical significance test on *S. cerevisiae* dataset

*P*-value	FFANE-XGB	FFANE-SVM
Bio2Vec	5.71E-12	1.84E-09
OLPP-SVM	1.79E-14	2.63E-16
SIFT-SVM	1.97E-10	3.63E-08
SNLF + QSO	1.30E-04	2.48E-03
AE-SVM	1.51E-09	1.25E-06
PSSM-SVM	3.04E-12	1.34E-13
TAGPPI	5.30E-07	1.07E-10
FFANE-SVM	1.17E-10	NA
FFANE-XGB	NA	1.17E-10

**Table 9:** Results of statistical significance test on *H. pylori* dataset

*P*-value	FFANE-XGB	FFANE-SVM
SIFT-SVM	1.24E-11	8.66E-04
Bio2Vec-Based	1.00E-03	5.62E-11
FFANE-SVM	7.11E-04	NA
FFANE-XGB	NA	7.11E-04

**Table 10:** Results of statistical significance test on *H. sapiens* dataset

*P*-value	FFANE-XGB	FFANE-SVM
AE-SVM	1.22E-09	6.91E-08
Bio2Vec-Based	2.73E-08	6.32E-04
SIFT-SVM	1.00E-09	9.00E-06
OLPP-SVM	4.22E-13	3.56E-13
FFANE-SVM	7.22E-10	NA
FFANE-XGB	NA	7.22E-10

## Conclusion

In this research, we introduced a novel approach called FFANE that leverages feature fusion in SAE for protein feature extraction. Following an exhaustive Grid Search to determine the optimal weighting coefficients for two types of information, we obtained multiple sets of feature vectors. Subsequently, we trained SVM to test the accuracy and selected the optimal alpha value. At the optimal alpha value, the FFANE’s node representation can be considered as accurately expressing node features. Moreover, we replaced the classifier with a more robust one, which typically requires longer training time compared to SVMs, but exhibits stronger classification capabilities.

The effectiveness of our proposed method is validated from several perspectives. Three classical datasets were used. By tuning the parameter alpha of our proposed method from zero to one that indicates the portion between the PPI profile and sequence profile, the best value of alpha was selected. Noted that setting alpha to zero or one cannot yield the highest prediction accuracy. When compared to the state-of-the-art methods, the performance of our proposed method demonstrated that it is promising for PPI prediction.

Besides, most state-of-the-art methods are dominated by deep learning models, with protein language models showing tremendous potential, like AlphaFold and ESM-2. However, it is worth noting that deep learning models often require powerful computational resources (such as CUDA core computing capability) and considerable effort for model debugging and training. In contrast, the FFANE algorithm has modest hardware requirements, offering greater flexibility and lower time costs. When incorporating new protein profiles, we can explore fusion learning, conduct testing and validation using SVM, and compare the results with benchmark tests based on SVM mentioned in state-of-the-art algorithm works to assess effectiveness.

In future work, there are some improvements to our proposed method. Firstly, the introduction of novel feature representation methods is viable, as a more precise numerical representation of protein profiles is crucial for minimizing noise and constructing an overall robust model. Secondly, there is room for improvement in the fusion methods employed for different features. Thirdly, with the enhancement of hardware computational capabilities and the reduction in computation costs, it becomes feasible to train more complex and powerful neural networks for deeper feature learning models, including protein language model.

## Methods

We developed a computational approach called FFANE to extract protein features. The proposed method integrates the Gaussian kernel similarity matrix and Levenshtein distance-based protein sequence similarities through weighted fusion, followed by Stacked Autoencoder (SAE) encoding learning, ultimately enabling accurate prediction of protein-protein interactions using machine-learning methodologies.

## Datasets

In the context of academic research, three distinct datasets were selected for analysis: the *Saccharomyces cerevisiae* (*S. cerevisiae*) dataset, the *Homo sapiens* (*H. sapiens*) dataset, and the *Helicobacter pylori* (*H. pylori*) dataset. The details of the datasets are listed in Table 11.

The *S. cerevisiae* dataset was curated from the core subset of interacting proteins sourced from the Database of Interacting Proteins (DIP) at https://dip.doe-mbi. ucla.edu/dip [35, 36]. Most protein pairs we collected exhibited pairwise sequence identities below the 40% threshold upon sequence alignment. 5594 pairs with positive interactions are obtained. Using sub-cellular localizations, 5,594 pairs with negative interactions are constructed, which results in accordance with the work in [35].

The *H. sapiens* dataset originated from the Human Protein References Database at https://hprd.org [37]. The PPI dataset comprises 8161 empirically validated PPIs spanning 2835 distinct human proteins. Rigorous data curation identified 3899 unique positive PPIs and 4262 negative PPIs after excluding self-interactions and duplicate instances.

The *H. pylori* dataset sought to unravel the molecular intricacies underlying the bacterium’s survival strategies and pathogenic tendencies [38]. Comprising 808 distinctive protein entities emblematic of *H. pylori*, positive and negative are 1,458. These interactions were categorized into distinct classes, considering the experimental evidence supporting each, including physical association, co-expression, and co-localization. Also, the processed dataset can be downloaded at https://github.com/YuBinLab-QUST/EResCNN/tree/main/Dataset.

**Table 11:** Detail of *S. cerevisiae*, *H. sapiens*, and *H. pylori* dataset

Dataset	Protein entity	Positive	Negative	Total No.
*S. cerevisiae*	2533	5594	5594	11,188
*H. sapiens*	2835	3899	4262	8161
*H. pylori*	808	1458	1458	2916

### Construction of protein similarity

Within the framework of our proposed methodology (also see phase 1 in Fig. 1), we amalgamate protein sequence details with interaction data, subsequently harnessing SAE to facilitate feature encoding and learning. To optimize the amalgamation of these information streams, prior to the fusion procedure, we employ tailored techniques that cater to the distinct attributes of protein interaction data and sequence information. More precisely, we employ a Gaussian kernel-based similarity metric for protein interaction data and utilize the Levenshtein distance metric for sequence information before the fusion process. Specifically, the Gaussian kernel is widely used in many fields for its efficiency in refining useful information from any input.

Let $G=(V,E)$ denotes the vertexes $V$ of proteins, as well as the edges $E$ representing the interactions between them. Given an adjacency matrix $A\in {\mathbb{R}}^{{n}_{protein}\times {n}_{protein}}$ of the PPI network with ${n}_{protein}$ proteins, the Gaussian kernel-based similarity value between the $i$-th protein $p\left(i\right)$ and $j$-th protein $p\left(i\right)$ is calculated as follow:

1 $${S}_{network}\left(p\right(i),p(j\left)\right)=exp(-{\gamma }_{r}{\Vert A\left(p\left(i\right)\right)-A\left(p\left(j\right)\right)\Vert}^{2}),$$

Where ${{\gamma }}_{r}$ denotes the Gaussian kernel bandwidth. Its definition is as follow:

2 $${{\gamma }}_{r}={\left[\left({\sum }_{i=1}^{n}{\Vert A\left(p\left(i\right)\right)?}^{2}\right)/n\right]}\Vert{-1}.$$

To construct sequence-based similarity, the Levenshtein distance metric was employed. The core idea of this algorithm is to calculate the similarity between two sequences according to making the fewest modification steps (insertions, deletions, and modifications) necessary to make the sequences identical [39, 40]. Here, a standard Python package is introduced to learn the similarity between proteins [41]. The latest release is Biopython 1.79, released on 3 June 2021 (https://biopython.org). The Biopython tool offers a series of bioinformatic analysis tools, including reverse complementation of DNA strings, searching for motifs in protein sequences, and others. Finally, a protein similarity matrix ${S}_{seq}$ can be obtained.

### Feature fusion matrix

Using a single feature type cannot reveal the potential mechanism in more depth. Therefore, it is a challenging task to improve efficiency by merging different types of features. Here, we propose fusing the structural and attributed information derived from the proteins’ interaction profile and sequence profile. The features fusion matrixes are computed and merged using the weighting method.

Given a Gaussian kernel-based network similarity matrix${S}_{network}{\mathbb{R}}^{{n}_{protein}\times {n}_{protein}}$ and a Levenshtein distance metric-based sequence similarity matrix ${S}_{seq}\in {\mathbb{R}}^{{n}_{protein}\times {n}_{protein}}$, the fusion matrix, is denoted as follow:

3 $$M=\alpha {S}_{network}+\left(1-\alpha \right){S}_{seq}$$

where each element in the matrix represents the proximity of transition from one protein to the others, so the matrix is also called a proximity matrix. Note that the parameter $\alpha$ ranges from 0 to 1.

Previous work Katz index focuses on emerging multiple proximity matrices with different orders, and more and more network embedding or node embedding methods like node2vec, DeepWalk, and LINE are developed to learn the node features based on the structural information [42, 43]. Not like these existing works by only using the limited interaction profile, our proposed method for fusing proximity matrixes aims to integrate two kinds of proteins including sequence profile and interaction profile of proteins. Such proximity matrix contains much node information that can be utilized in protein feature representation [44].

### Stacked autoencoder for node embedding

**Fig. 14:**
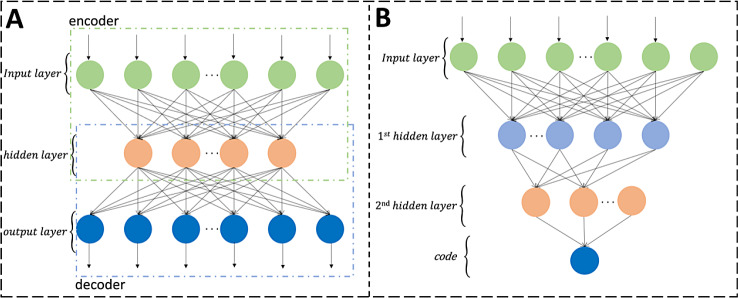
Schematic of the architecture of a basic AE and a SAE. (**A**) A basic AE with one input layer, one hidden layer, and one output layer. (**B**) A SAE for node embedding with one input layer, two hidden layers, and one output layer

The constructed fusion matrix combines the node attribute with the structural information, also called the initial information fusion matrix. More notably, the dimension of the initial information fusion matrix is N, where N represents the number of proteins, while the constructed feature vector is 2*N. Excessively high dimensions pose a catastrophic challenge to model training, often resulting in prolonged training times or even training failures. Furthermore, Such a matrix is informative but inefficient for model training and it still needs to be refined for better downstream learning tasks. SAE as a non-linear dimensionality reduction technique is widely used for feature learning of nodes with raw features. It can generate the node embedding by mapping the raw sequence or coding into a new feature space with lower dimensions but higher efficiency. The definition of SAE is as follows [45]:

SAE is a deep learning model that constructs a deep neural network by stacking multiple hidden layers, leveraging the concept of AE. Each hidden layer focuses on learning different levels of abstract features from the data, progressively enhancing the representation capability of the features. A basic AE, illustrated in Fig. 14(A), can be defined in two parts: encoder and decoder. Given an original input dataset $x\in {\mathbb{R}}^{n}$, the goal of encoder is to map $x$ into encoding feature $h\in {\mathbb{R}}^{d}$ by using a transformation matrix ${W}_{encoder}\in {\mathbb{R}}^{d\times n}$, where $d$ denotes the number of neurons in hidden layer. Then, the goal of decoder is to obtain the constructed feature $\stackrel{\sim}{x}$ by using a transformation matrix ${W}_{decoder}\in {\mathbb{R}}^{n\times d}$ on $h$. The definitions of encoder and decoder are as follows:

4 $$h=\sigma ({W}_{encoder}x+{b}_{encoder})$$

5 $$\stackrel{\sim}{x}=\sigma ({W}_{decoder}h+{b}_{decoder})$$

where ${b}_{encoder}$ and ${b}_{decoder}$ are the parameters in the encoder and decoder, and $\sigma (\cdot)$ is the activation function. SAE learns the nodes’ features without the corresponding labels, in which the parameters ${W}_{encoder}$,${W}_{decoder}$,${b}_{encoder}$ and ${b}_{decoder}$ are corrected and optimized by minimizing the reconstruction error between input and output via a loss function and gradient descent algorithm. The loss function can be defined as follows:

6 $${F}_{loss}=\frac{1}{N}\sum _{i}{\Vert{x}_{i}-{\stackrel{\sim}{x}}_{i}\Vert}_{2}^{2}$$

where $N$ denotes the number of samples. Further,${F}_{loss}$ can be formulated by encoder $f$ and decoder $g$ as:

7 $${F}_{loss}=\frac{1}{N}\sum _{i}{\Vert{x}_{i}-g\left(f\right({x}_{i}\left)\right)\Vert}_{2}^{2}$$

In this study, we investigated SAE, comprising two hidden layers. The architecture of SAE is depicted in Fig. 14(B). In our SAE feature learning setup, as illustrated in Fig. 14(B), the SAE architecture utilized lacks the decoder component, employing only the encoder for the purposes of feature reconstruction learning and dimensionality reduction. Specifically, the hidden layers consist of two layers, enhancing features progressively, ultimately leading to output at the output layer. The specific parameters are as follows: N (input layer), 1024 (hidden layer), 512 (hidden layer), 128 (output layer).

### Construction of support vector machine classification model

As all nodes in the heterogeneous graph are projected in a continuous vector space by using SAE, support vector machine (SVM) classifier can cooperate well with such continuous vector features to discriminate positive ones and negative ones by an optimal hyperplane.

Given a constructed feature set $x\in {\mathbb{R}}^{n\times d}$ with $n$ samples and $d$ dimensions as a set of protein-target data, each sample ${x}_{i}^{{\prime }}$ of $x$ tagging to a class $y$ can be denoted as

8 $${y}_{i}=class({x}_{i}^{{\prime }}=\left\{{x}_{ij}^{{\prime }},{x}_{ij}^{{\prime }},\cdots,{x}_{ij}^{{\prime }}\right\})$$

where ${x}_{ij}^{{\prime }}$ denote $j$-th column feature of ${x}_{i}^{{\prime }}$. As the optimal hyperplane in SVM needs to be generated to classify samples accurately based on the input training set, there are various kernels for different scenarios such as linear, sigmoid kernels, polynomial, and Gaussian radial basis function (RBF). Here, RBF kernel is selected, and the definition is as follows:

9 $$k\left({x}_{i},{x}_{j}\right)=\text{e}\text{x}\text{p}(-{\gamma }{\Vert{x}_{i}-{x}_{j}\Vert}^{2})$$

where ${\gamma }$ is an important coefficient of the kernel function, i.e. kernel bandwidth. In practice, a slack variable $\xi$ must be introduced to fix the noise in feature set, which can loosen the constrains:

10 $${y}_{i}\left(\langle w,{x}_{i} \rangle +b\right)\ge 1-{\xi }_{i}$$

where $w$ and $b$ are the parameters adjusted by SVM for decision margin, and $i$ ranges from 1 to $n$. To obtain the optimal result, the objective function is defined as follow:

11 $$\text{m}\text{i}\text{n}(\frac{{\Vert w \Vert}^{2}}{2}+C\sum _{i=1}^{n}{\xi }_{i})$$

where $C$ is the important parameter for penalty constant of training error. In this study, SVM classifiers were implemented by using the libSVM tool.

### Implementation

The FFANE is a two-part process that involves constructing an initial information fusion matrix and utilizing the Stacked Autoencoder (SAE) for node representation. To construct the initial information fusion matrix, an alpha parameter must be established, which we evaluate between 0 and 1 with intervals of 0.125. The SAE is implemented using TensorFlow in Python, with a layered architecture consisting of N (input layer), 1024 (the 1st hidden layer), 512 (the 2nd hidden layer), and 128 (output layer). Based on our experience, the maximum number of epochs, batch size and learning rate of Adam optimizer are set to 100, 32, and 0.001, respectively. EarlyStopping is utilized with a patience of 30. Additionally, the mean squared error loss function is used.

## Data Availability

The datasets and python code supporting the findings of this study are available at https://github.com/StacyMYCao/FFANE.
